# Diet–Oral Microbiota Interactions and Salivary Biomarkers of Nutritional Health: A Narrative Review

**DOI:** 10.3390/nu18030396

**Published:** 2026-01-25

**Authors:** Liliana Anchidin-Norocel, Andrei Lobiuc, Mihai Covasa

**Affiliations:** 1 College of Medicine and Biological Science, Stefan cel Mare University of Suceava, 720229 Suceava, Romania; andrei.lobiuc@usm.ro (A.L.); mihai.covasa@belmont.edu (M.C.); 2 Thomas F. Frist, JR. College of Medicine, Belmont University, Nashville, TN 37212, USA

**Keywords:** saliva, nutrients, oral microbiome, heavy metals toxicity, dysbiosis

## Abstract

Diet plays a central role in shaping the composition and metabolic activity of the oral microbiota, thereby influencing both oral and systemic health. Disturbances in this delicate host–microbe balance, triggered by dietary factors, smoking, poor oral hygiene, or antibiotic use, can lead to microbial dysbiosis and increase the risk of oral diseases such as periodontitis, as well as chronic systemic disorders including diabetes, cardiovascular disease, Alzheimer’s disease, and certain cancers. Among dietary contaminants, exposure to toxic heavy metals such as cadmium (Cd), lead (Pb), mercury (Hg), nickel (Ni), and arsenic (As) represents an underrecognized modifier of the oral microbial ecosystem. Even at low concentrations, these elements can disrupt microbial diversity, promote inflammation, and impair metabolic homeostasis. Saliva has recently emerged as a promising, non-invasive biofluid for monitoring nutritional status and early metabolic alterations induced by diet and environmental exposures. Salivary biomarkers, including metabolites, trace elements, and microbial signatures, offer potential for assessing the combined effects of diet, microbiota, and toxicant exposure. This review synthesizes current evidence on how diet influences the oral microbiota and modulates susceptibility to heavy metal toxicity. It also examines the potential of salivary biomarkers as integrative indicators of nutritional status and metabolic health, highlights methodological challenges limiting their validation, and outlines future research directions for developing saliva-based tools in personalized nutrition and precision health.

## 1. Introduction

The human oral cavity harbors more than 700 microbial species, classified into 185 genera and 12 major bacterial phyla. Of this diverse community, approximately 54% of species have been fully characterized, 14% have been cultivated but remain unnamed, and about 32% are known only from DNA-based signatures (“phylotypes”) and have not yet been cultured or fully described [1,2]. As the entry point of the digestive tract, the oral microbiome influences nutrient absorption, metabolism, and immune function [3]. Dysbiosis of this microbial ecosystem is associated not only with oral diseases but also with systemic conditions such as diabetes, cardiovascular disease, Alzheimer’s disease, cancer, and increased mortality risk [4,5].

Dietary patterns strongly influence the composition and function of the oral microbiota [6]. Studies comparing distinct dietary groups, including vegetarians, individuals following a Western diet, traditional farmers, and hunter-gatherers, demonstrate marked differences in oral microbial communities [7,8,9,10]. Using 16S rRNA sequencing, researchers have shown that the relative abundance of key taxa such as *Neisseria* and *Haemophilus* varies substantially according to dietary habits. Hunter-gatherer populations, who consume larger amounts of animal protein, display higher levels of several oral pathogens [11], whereas vegetarian diets induce broad shifts across multiple taxonomic levels, including changes in both oral pathogens and respiratory-associated microbes. Thus, diet also shapes microbial function; for instance, dietary transitions have been linked to adaptations such as vitamin B5 autotrophy and urease-mediated pH regulation [12].

Despite progress in oral health research, conventional diagnostic approaches remain retrospective and limited in their ability to detect early or subclinical disease, emphasizing the need for non-invasive, reproducible biomarkers capable of identifying active biological processes before irreversible tissue damage occurs [13]. Saliva has emerged as an especially promising diagnostic medium because it can be collected easily and non-invasively while reflecting both local oral conditions and systemic physiology [14]. However, the translational application of salivary biomarkers is currently constrained by substantial methodological heterogeneity, including differences between stimulated and unstimulated saliva collection, circadian variation, sample handling, and analytical platforms [15,16,17]. In parallel, advances in oral microbiome research have shifted the field from a pathogen-focused view to a broader ecological framework, recognizing microbial community imbalance, rather than specific pathogens alone, as a central driver of inflammation and tissue destruction [18].

Recent findings indicate that the oral ecosystem can display dynamic responses to physiological and environmental influences, although longitudinal studies suggest that, in the absence of disease or changes in oral hygiene, oral microbial communities remain relatively stable over time [19,20]. A study conducted by Sarkar et al. demonstrated that the salivary microbiome and salivary cytokines (IL-1β, IL-6, IL-8) exhibit circadian oscillations in healthy adults, with specific bacterial taxa fluctuating in synchrony or anti-phase with inflammatory markers. These observations underscore the potential of saliva as a non-invasive tool for monitoring temporal biological patterns in oral and systemic health [21].

Despite its remarkable diversity, the oral microbiome remains underexplored in relation to its role in maintaining health and its involvement in non-infectious diseases [22]. Alterations in oral microbial composition have been linked to obesity, Type 2 diabetes, and Metabolic Syndrome, suggesting that obesity-related changes may drive dysbiosis and contribute to metabolic dysfunction [23]. Salivary biomarkers have increasingly gained attention as potential indicators of metabolic status [24,25,26,27]. Their non-invasive nature and ability to reflect both local and systemic physiology make them promising tools for detecting early metabolic alterations and assessing individual susceptibility to metabolic dysfunction [28,29].

Although the current literature increasingly recognizes the role of diet in shaping the oral microbiota and influencing metabolic health, one important factor remains largely overlooked: the impact of dietary heavy metals on oral dysbiosis. Food-borne exposure to toxic metals, well known for disrupting the gut microbiome and metabolic pathways [30,31,32,33], is rarely integrated into studies of the oral microbiome, despite emerging evidence that these contaminants can alter microbial composition, promote oral pathogens, and modulate inflammatory and metabolic responses [34]. This conceptual gap is also evident in the bibliometric analysis where diet-, microbiota-, and metabolism-related terms are well represented, while heavy metals are almost entirely absent, highlighting a clear deficiency in the current research landscape.

### Literature Search Strategy

This narrative review was informed by a structured, non-systematic literature search conducted using PubMed, Scopus, and Web of Science, covering studies published primarily between 2000 and 2025. Search terms included combinations of keywords such as “oral microbiome,” “diet,” “nutrition,” “saliva,” “salivary biomarkers,” “metabolic health,” “obesity,” and “heavy metals.” Bibliometric mapping and keyword network analyses were used to identify dominant research themes and knowledge gaps relevant to the aims of this review.

**Figure 1:**
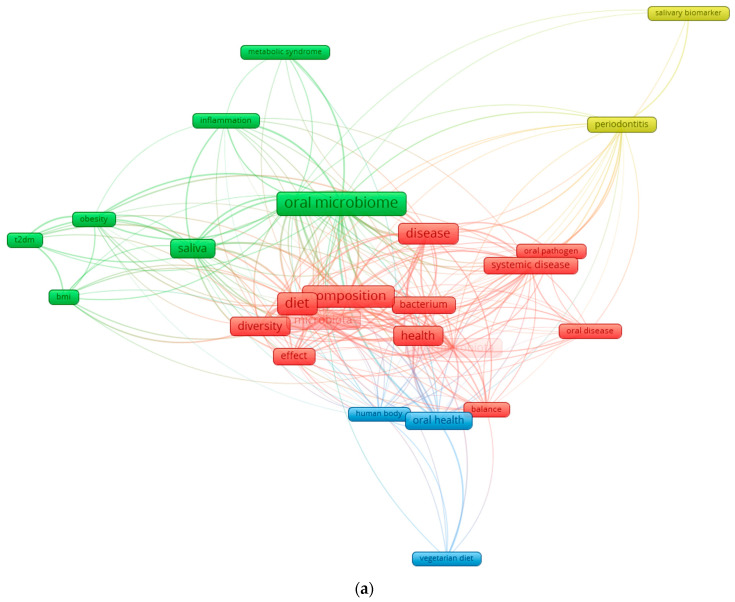

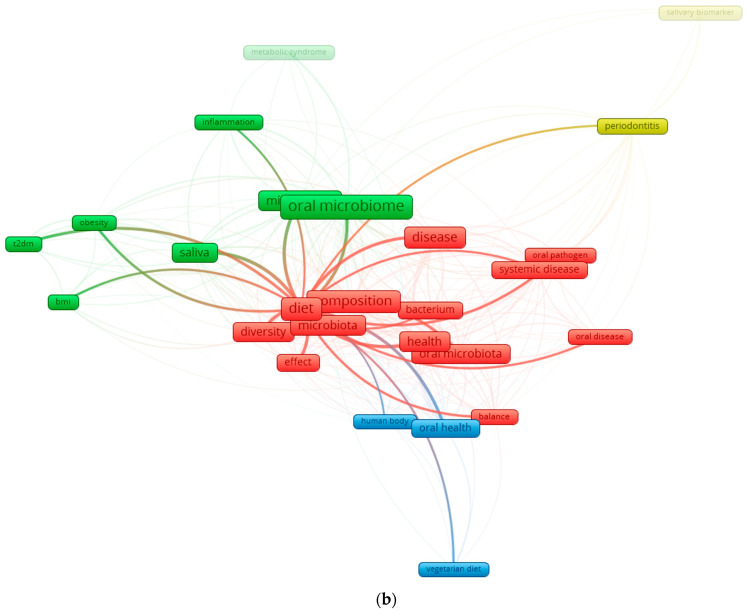
Keyword co-occurrence network in the field of diet–oral microbiota research. (**a**) illustrates the global keyword co-occurrence network derived from recent literature on oral microbiota, diet, and health. Node size reflects the frequency of each keyword, while the color gradient represents the average publication year (from dark blue for earlier terms to yellow for more recent ones). Core concepts such as oral microbiome, diet, microbiota, saliva, health, and disease form the central structure of the network, indicating their dominant role in the field. Additional clusters highlight established themes (e.g., periodontitis, systemic disease) as well as emerging topics such as vegetarian diet and metabolic syndrome. (**b**) focuses exclusively on the connections of the keyword “diet.” This subnetwork isolates all terms directly linked to diet, revealing its relationships with microbiota composition, saliva, diversity, oral health, obesity, T2DM, and body mass index (BMI). The high density of links underscores the central role of dietary patterns in shaping oral microbial ecology and in modulating systemic metabolic pathways. By separating the global and diet-specific networks, the figure highlights both the broader research landscape and the specific influence of diet within it.

In this context, the primary objective of this review is to synthesize and critically appraise current evidence on how diet acts as a major upstream determinant of oral microbiota composition and function. Building on this foundation, we examine how diet-associated microbial alterations are reflected in salivary biomarkers and explore their potential relevance for systemic metabolic health. Secondary topics, including the role of dietary and environmental heavy metals, are discussed as emerging and exploratory factors that may further modulate oral microbial ecology and salivary biomarker profiles. By establishing this hierarchical framework, diet → oral microbiota → salivary biomarkers → systemic implications, this review aims to provide a coherent integrative perspective, highlight key knowledge gaps, and identify translational challenges relevant to personalized nutrition and precision health. In this review, the terms “eubiosis” and “dysbiosis” denote balanced versus disrupted microbial community states, respectively. The term “healthy diet” refers to fiber-rich, minimally processed dietary patterns, whereas “Western diet” denotes dietary patterns high in refined carbohydrates, saturated fats, and ultra-processed foods. Figure 1 illustrates the current research landscape and underscores the need for this integrated perspective.

## 2. Oral Microbiota Composition

At birth, the oral microbiota is initially composed of a limited number of early colonizing genera, most commonly including *Streptococcus* and other facultative anaerobes; however, the relative abundance and taxonomic composition vary considerably depending on population, geographic region, delivery mode, feeding practices, and methodological approaches. As infancy progresses, additional genera such as *Veillonella*, *Fusobacterium*, and *Lactobacillus* may increase in abundance, although this developmental trajectory is not uniform across studies [35,36,37]. *Staphylococcus* peaks around three months of age and then declines, concurrent with a rise in *Gemella*, *Granulicatella*, *Haemophilus*, and *Rothia* species. With tooth eruption, the oral ecosystem undergoes a major ecological transition, gradually incorporating higher abundances of *Fusobacteriota*, *Synergistetes*, *Tenericutes*, *Saccharibacteria* (TM7), and *SR1* phyla as individuals progress toward adulthood [38].

The mature human oral cavity hosts a highly diverse microbial ecosystem composed of bacteria, archaea, fungi, and viruses, organized within complex biofilms that contribute to oral homeostasis [39,40]. Most bacterial members belong to the major phyla *Actinobacteria*, *Bacteroidetes*, *Chlamydia*, *Euryarchaeota*, *Fusobacteria*, *Firmicutes*, *Proteobacteria*, *Spirochaetes*, and *Tenericutes*, with additional, low-abundance groups such as *Chloroflexi*, *Chlorobi*, *GN02*, *Synergistetes*, *SR1*, *TM7*, and *WPS-2*—are also present. Among these, *GN02, SR1*, and *TM7* are classified within the Candidate Phyla Radiation (CPR), whose members influence the structural organization and functional dynamics of the oral microbial network, with implications for diseases such as periodontitis and halitosis [41]. Microbial species support oral and systemic health by preventing pathogenic colonization, contributing to nutrient metabolism, and regulating immune responses [42]. However, when this ecological balance is disrupted, the resulting dysbiosis can promote the development of both oral diseases and systemic disorders [43,44].

## 3. Diet Influence on Oral Microbiota

Oral health is influenced by various internal and external factors throughout life [45]. The adaptability of the oral cavity is shaped by host-related factors such as genetics, age, immune function, and lifestyle, as well as environmental factors including diet, pH, gingival crevicular fluid, and saliva [46,47], to modulate the diversity and composition of the oral microbiota [48]. It should be noted that antibiotic exposure, use of antiseptic mouth rinses, tobacco consumption, oral disease, and oral hygiene practices represent the primary drivers of oral microbiome variation, whereas diet acts predominantly as a modulator of microbial structure [20].

Recent evidence consolidates diet as one of the strongest modulators of oral microbial structure. Metin et al. [49] reviewed clinical evidence suggesting that healthier dietary patterns, characterized by higher intake of plant-based foods and fiber and lower consumption of refined sugars and processed foods, are associated with improved oral and periodontal health outcomes, including lower plaque accumulation and gingival inflammation. Although microbial taxa were not directly assessed, these findings indirectly support a link between diet quality and a more favorable oral environment. Similarly, Angarita-Díaz et al. [50] synthesizing studies comparing high- versus low-sugar intake through 16S rRNA sequencing, found that high sugar consumption significantly reduces microbial diversity and shifts community composition toward *Streptococcus*, *Scardovia*, *Veillonella*, *Rothia*, *Actinomyces*, and *Lactobacillus*. Specifically, frequent sugar exposure tended to favor acidogenic/aciduric bacteria at the expense of others, indicating a diet-induced dysbiosis rather than a stable, balanced microbiome [51]. Conversely, dietary fiber appears to exert beneficial effects. Kondo et al. [52] demonstrated that an eight-week high-fiber, low-fat dietary intervention in metabolically at-risk individuals significantly improved periodontal parameters, including reductions in probing depth, clinical attachment loss, and bleeding on probing, alongside improvements in systemic metabolic markers (body weight, HbA1c, hs-CRP). Although microbiota composition was not directly assessed, the findings suggest that fiber-rich diets can indirectly support a more balanced oral ecosystem by reducing inflammation and improving host metabolic status .

Antioxidants also contribute to maintaining oral ecological stability. Malcangi et al. [53] reports that dietary or supplemental intake of natural antioxidants supports endogenous antioxidant systems, protecting oral mucosal tissues from oxidative stress. Natural antioxidants modulate immune responses and the redox environment of the oral cavity, thereby helping limit the detrimental effects of pathogenic biofilms and chronic inflammation .

Additional functional foods and nutrients, including mangosteen (rich in xanthones and vitamin C), vitamin D, omega-3 polyunsaturated fatty acids, and polyphenols, have been investigated as potential adjuncts for oral health. Available evidence, largely derived from small clinical trials and in vitro or short-term interventions, suggests possible benefits for oral inflammatory status and microbial balance; however, robust microbiome-based clinical data remain limited due to short trial durations and small sample sizes.

Fermented lingonberry juice used as a mouthwash for six months has demonstrated reductions in pathogenic species and increases in beneficial bacteria such as *Lactobacillus* [54]. Plant-based diets, including vegetarian and vegan patterns, may reduce periodontitis risk through higher fiber intake and lower pro-inflammatory saturated fats, though vegan diets may increase susceptibility to dental erosion and caries due to lower calcium, vitamin B12 intake, and reduced salivary pH [55,56,57]. Dietary influences on the oral microbiota also include the role of fermented foods and probiotic-containing products [58,59]. Nutrients introduced through meals act as substrates for oral bacteria, shaping which species can thrive in the oral cavity. Fermented foods enriched with *Lactobacillus* and *Bifidobacterium* have well-established benefits for gut microbial balance and systemic health, and *Lactobacillus* species in particular can inhibit cariogenic pathogens such as *Streptococcus mutans* [60]. Experimental evidence further shows that fermented Japanese mugwort (Yomo gyutto) increases salivary secretion and alters the oral microbiota in mice, suggesting that certain fermented foods may exert dual effects on both oral and gut microbial communities [61].

Additionally, patterns of habitual food intake appear to influence the salivary microbiota. Hansen et al. [62] in a study of 160 healthy adults comparing vegans and omnivores, found subtle but significant differences in beta-diversity and in specific microbial taxa. Vegans exhibited higher abundances of commensals such as *Neisseria subflava*, *Haemophilus parainfluenzae*, and *Rothia mucilaginosa*, whereas omnivores displayed higher levels of *Prevotella melaninogenica* and *Streptococcus* species [62]. Dietary components including medium-chain fatty acids, marine mono- and polyunsaturated fatty acids, and dietary fiber were associated with both taxonomic and predicted functional variation, and several salivary bacteria correlated with systemic inflammatory markers, linking dietary patterns, oral microbiota, and host inflammation [63,64].

### Dietary and Environmental Metal Exposure and Oral Microbiota Interactions

In the context of nutrition, dietary and environmental metal exposure can be conceptualized as an extension of diet-related inputs, reflecting not only food choices but also food quality, processing, and environmental contamination along the food chain. Beyond macronutrients and bioactive dietary compounds, environmental and dietary contaminants such as heavy metals have emerged as additional modulators of the oral microbiome [65,66]. From a dietary perspective, metal exposure reflects both food-related and environmental pathways. Dietary intake through contaminated food and drinking water represents a major chronic exposure route in the general population, whereas occupational or environmental exposures (e.g., air, soil) may contribute more substantially in specific settings (e.g., contamination of vegetables and irrigation water from polluted environments) [67]. The relative contribution of these pathways depends on dietary patterns, food sourcing, processing, and local environmental conditions. These metals can enter the body not only through smoking or environmental pollution but also through contaminated foods such as crops grown in polluted soils, cereals, fish and seafood, drinking water, and certain processed products [68,69,70]. Heavy metal exposure has been linked to microbiome-mediated conditions such as caries and gingival inflammation [71]. Salivary concentrations of metals such as antimony, arsenic, and mercury are associated with distinct microbial shifts; notably, elevated antimony correlates with higher levels of *Lactobacillus* spp., a genus strongly associated with acidogenic metabolism and caries development [72,73]. Exposure to additional metals, including chromium, nickel, and copper, has been associated with shifts in the relative abundance of genera such as *Capnocytophaga*, *Neisseria*, *Aggregatella*, *Streptococcus*, *Campylobacter*, *Selenomonas*, and *Prevotella*, reflecting structural alterations in the microbial community [74]. The salivary microbiome responds to environmental exposures such as metals, which bacteria need for metabolism but which can become toxic at elevated levels [75]. Because of this dual role, metal exposure can alter microbial composition and potentially affect oral health [76]. Importantly, most available evidence reflects associative findings, and relatively few studies distinguish between acute high-dose exposure and chronic low-dose exposure in otherwise healthy individuals [74,77]. Chronic dietary exposure through contaminated drinking water, soil-derived crops, seafood, or certain processed foods may induce subtle but persistent shifts in oral microbial communities without overt clinical disease [78]. Dietary context may further modulate these effects, as processed foods may increase exposure to contaminants and reduce microbial resilience, whereas whole-food diets may partially buffer metal-associated dysbiosis. Population-level observations from environmentally contaminated regions suggest that long-term metal exposure can disrupt microbial community structure and functional balance, yet studies integrating environmental contamination, dietary patterns, and oral microbiome profiling remain scarce [79]. Addressing these gaps will be essential for clarifying dose–response relationships and translational relevance.

**Figure 2:**
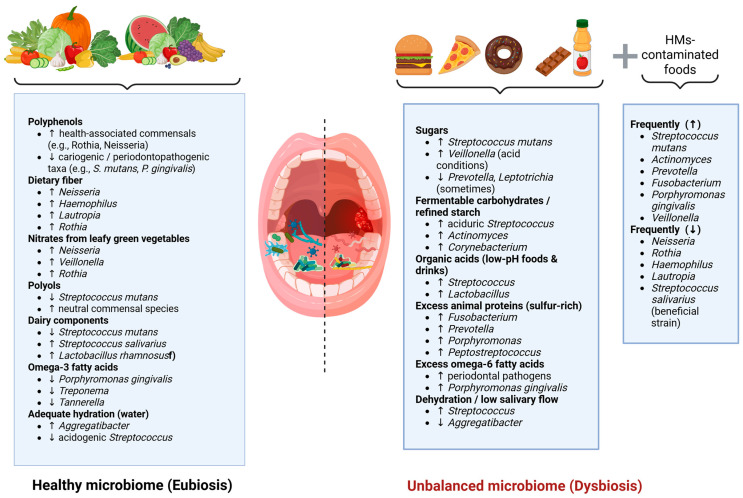
Conceptual overview of dietary and environmental factors associated with modulation of the oral microbiome, based on the evidence discussed throughout the manuscript. Beneficial dietary factors (**left**) promote eubiosis by supporting health-associated oral bacteria, whereas sugars, fermentable carbohydrates, acidic foods, excessive animal proteins, omega-6 fatty acids, and low salivary flow (**right**) shift the microbiota toward dysbiosis. Heavy-metal-contaminated foods additionally contribute to pathogenic microbial profiles. Arrows indicate the direction of microbial changes (increase or decrease), while dashed lines represent the transition between eubiosis and dysbiosis.

Unlike blood or urine, where reference guidelines exist, there are no established standards for interpreting salivary metal levels. Still, saliva is a promising biomarker for assessing metal exposure, especially when the oral cavity is the main target of toxicity. Although some studies have measured metals like lead, mercury, iron, magnesium, and zinc in saliva, research in this area remains limited [80]. A summary of how dietary components shape the oral microbiome is presented in Figure 2.

## 4. Oral Microbiota and Nutritional Metabolism

The oral microbiota can influence the gastrointestinal tract through several mechanisms [81,82,83]. Oral bacteria may directly colonize the gut, disrupt microbial balance and affect digestive processes, including key functions such as butyrate production [48,83]. Periodontal pathogens can also enter the bloodstream, disseminate systemically, and contribute to diseases such as colorectal cancer; however, the frequency and clinical relevance of this process in humans remain unclear. In addition, microbial metabolites originating in the oral cavity may circulate through the blood, triggering low-grade inflammation and promoting chronic digestive disorders. Increasing evidence supports these pathways, suggesting that oral bacteria play a significant role in gut dysbiosis and systemic health [84,85].

Obesity is characterized by chronic low-grade inflammation that links it to metabolic diseases. This obesity-associated inflammation often referred to as “metainflammation” is especially evident in adipose tissue and involves inflammatory pathways that regulate metabolic homeostasis [86]. Supporting evidence includes findings that anti-inflammatory therapies improve metabolic outcomes, while weight loss reduces systemic inflammation [87,88]. The gut microbiome shows characteristic alterations in obesity, but growing data indicate that oral dysbiosis may also contribute to inflammatory processes in obesity, promoting metainflammation within adipose tissue and aggravating metabolic dysfunction [89].

Although the effects of diet and nutritional status on the gut microbiome and metabolome are well documented, their impact on the oral microbiome remains less thoroughly explored [90,91]. Nonetheless, existing evidence shows clear differences in oral microbial composition between obese and non-obese individuals, suggesting a potential role of oral bacteria in obesity [92,93]. Many dietary components, macronutrients, micronutrients, and pre/probiotics, as well as dietary patterns such as Mediterranean or low-carbohydrate diets, are known to influence gut microbiota and disease risk, but considerably less is known about how these factors shape the oral microbiome [94].

This knowledge gap is notable because diet is a major determinant of oral health and plays a crucial role in the development of dental caries and periodontal disease (PD). PD affects 20–50% of the global population and, in susceptible individuals, may contribute to or exacerbate systemic diseases such as cardiovascular disease and diabetes. These conditions also reduce quality of life and impose substantial economic burdens on healthcare systems [95].

Burcham et al. [96] profiled the oral microbiome of adults (20–57 years) and children (8–9 years) using 16S rRNA gene sequencing. Children exhibited higher evenness and Shannon diversity compared with adults. In adults, oral hygiene habits were the strongest determinants of microbiome variation, whereas in children, weight status and biological sex showed significant associations. The genus Treponema was more frequently detected in adults who had not visited a dentist recently and in obese children. Moreover, individuals from the same family shared more similar oral microbiomes than unrelated individuals, suggesting the combined influence of shared environment and behaviors, alongside potential host genetic contributions .

Yue et al. [97] reported that the oral microbiome produces and modifies various fatty acids, including short-chain fatty acids (SCFAs), which can influence the local oral environment. Animal studies show that oral inoculation with *Porphyromonas gingivalis* increases total free fatty acid (FFA) levels in tongue tissue and plasma and alters plasma FFA profiles, potentially via upregulation of de novo fatty-acid synthesis pathways. However, there is currently no definitive human evidence demonstrating that these microbial metabolites enter systemic circulation or directly regulate plasma FFA levels. Because elevated FFAs contribute to insulin resistance and are consistently observed in type 2 diabetes mellitus (T2DM), it is plausible that oral microbiome–derived metabolites may indirectly exacerbate insulin resistance by influencing FFA metabolism . The same authors further demonstrated that the oral microbiome can aggravate high-fat-diet (HFD)-induced insulin resistance. Oral inoculation with periodontal pathogens (*P. intermedia*, *F. nucleatum*, *P. gingivalis*) worsened insulin resistance and glucose intolerance associated with HFD, partly through antibody responses directed against *P. gingivalis LPS.* Additional experiments showed that *P. gingivalis* can increase insulin secretion and HOMA-IR even without elevating circulating FFAs, suggesting multiple mechanisms of metabolic disruption. Moreover, oral bacteria can increase serum branched-chain amino acids (BCAAs), activating the mTOR–S6K1 pathway and impairing insulin signaling. Overall, the oral microbiome may promote HFD-induced insulin resistance through immune-mediated pathways and microbial metabolites, although validating these findings in humans will require more advanced and integrative models [97]. When the association between salivary microbiome composition and metabolic syndrome in adults was examined, Silva et al. identified specific microbial alterations correlating with clinical parameters of metabolic syndrome, suggesting that the salivary microbial profile may serve as a potential non-invasive biomarker for metabolic status. Collectively, these findings support the value of the oral microbiome as an accessible indicator of systemic metabolism and cardiometabolic risk [23].

## 5. Salivary Biomarkers of Nutritional Health

Nutritional status reflects an individual’s health condition as determined by the intake, absorption, and utilization of nutrients [98]. Its assessment is traditionally complex, requiring a combination of anthropometric measurements, clinical examination, dietary evaluation, blood analyses interpreted alongside medical history, and consideration of environmental factors that influence eating behaviors [99]. Deviations from expected values, such as abnormal biochemical markers or insufficient nutrient intake, may signal risks of malnutrition, iron-deficiency anemia, osteoporosis, or type 2 diabetes if timely nutritional interventions are not implemented [100]. Gathering accurate dietary information remains challenging. Common dietary assessment tools, such as food frequency questionnaires, 24-h recall, and food diaries, are subjective, prone to recall bias, and often affected by errors in portion estimation [101]. In this context, dietary biomarkers can address many of these limitations by providing objective indicators that complement self-reported data. Biomarkers offer a more reliable link between nutrient intake, nutritional status, and disease risk, and they are increasingly used in the detection, monitoring, and diagnosis of various health conditions [102].

Saliva contains a diverse repertoire of biomolecules, including electrolytes, hormones, cytokines, oxidative stress markers, enzymes, and microbial products [103]. Recent studies demonstrate that salivary biomarkers can reflect both nutritional imbalances and metabolic dysfunction [104]. In inflammatory bowel disease (IBD), for example, salivary profiles encompassing oxidative stress markers, inflammatory cytokines, microRNAs, calprotectin, PSMA7, α-amylase, and antioxidant enzymes display distinct patterns across disease activity, saliva type (stimulated vs. unstimulated), and IBD subtype. Elevated IL-1β, α-amylase, and malondialdehyde (MDA), along with reduced glutathione and FRAP, have been associated with active disease. Moreover, salivary cytokines and secretory IgA correlate with the abundance of IBD-associated oral taxa such as *Prevotella*, *Haemophilus*, *Streptococcus*, and *Veillonella*, underscoring the diagnostic value of integrating salivary biomarkers with microbiome profiling [105]. In sports and metabolic physiology, salivary biomarkers have shown similar promise. Campos et al. [106] reported that saliva can reliably capture fluctuations in immune, endocrine, oxidative stress, and muscle-damage biomarkers, although the authors emphasized the need for methodological standardization and individualized reference frameworks before broad clinical application .

Additional evidence supports the relevance of specific salivary analytes for nutritional monitoring. For example, Logan et al. identified several salivary biomarkers with strong potential for reflecting nutritional status and dietary intake, including glucose, vitamin D, calcium, total antioxidant capacity (TAC), nitrate/nitrite, and fluoride. Salivary glucose correlated with serum glucose in individuals with type 2 diabetes; salivary calcium was higher in post-menopausal women, particularly those with reduced bone mineral density; and salivary vitamin D showed promise in reflecting serum vitamin D levels in healthy volunteers. Nonetheless, the evidence remains limited and heterogeneous, and salivary markers cannot yet fully replace traditional nutritional or dietary assessments [102,107].

Saliva is also emerging as a useful medium for assessing oxidative stress. Biomarkers such as vitamin C, MDA, α-amylase, and antioxidant enzymes have been shown to reflect exercise-induced oxidative stress, suggesting that salivary assays may provide a practical alternative to blood-based redox monitoring. Although research in this area remains preliminary, existing findings highlight saliva’s utility in capturing diet- and lifestyle-related physiological changes [108].

Salivary inflammatory and metabolic biomarkers have further demonstrated diagnostic potential in endocrine disorders. Opydo-Szymaczek et al. [109] reported that TNF-α, IL-6, IL-1β, uric acid, and testosterone exhibited high diagnostic accuracy for polycystic ovary syndrome (PCOS), with pronounced elevations even among normal-weight adolescents. These results indicate that salivary biomarkers can detect low-grade inflammation and androgen dysregulation independent of adiposity, supporting their use in early, non-invasive screening of metabolic–inflammatory disturbances . Similarly, a recent systematic review revealed that individuals with metabolically unhealthy obesity (MUO) consistently exhibit increased salivary concentrations of inflammatory and oxidative stress biomarkers, including 8-OHdG, IL-6, IL-8, resistin, TNFR1, PTX-3, AEA, OEA, TNF-α, and sICAM-1, suggesting that saliva may serve as an accessible indicator of obesity-related metabolic dysfunction and cardiometabolic risk [24]. Finally, salivary markers of carbohydrate and lipid metabolism show strong translational potential. In individuals with type 1 diabetes, salivary glucose, triglycerides, and cholesterol correlate significantly with corresponding serum levels, indicating that saliva can partially reflect systemic metabolic disturbances. These findings highlight salivary glucose and lipid markers as promising adjuncts to conventional blood-based assessments for monitoring glycemic control and dyslipidemia [110].

## 6. Integration of Diet, Oral Microbiota and Salivary Biomarkers in Nutritional Health Evaluation

Integrating information derived from diet, the oral microbiota, and salivary biomarkers offers a comprehensive framework for understanding how nutritional factors shape metabolic and inflammatory processes within the oral cavity [93]. Dietary intake directly modulates the availability of metabolic substrates for oral bacteria, driving shifts in microbial community structure and altering the production of key metabolites such as short-chain fatty acids (SCFAs), lactate, volatile compounds, and free fatty acids [46]. These microbiota-mediated changes are subsequently mirrored in the salivary biochemical profile through measurable variations in metabolic, inflammatory, and antioxidant markers [111]. Consequently, saliva emerges as an integrative biological matrix capable of capturing both nutritional status and the host’s biological responses to dietary exposures.

Recent advances in salivary metabolomics further demonstrate that diet-induced alterations in oral microbial activity leave detectable metabolic signatures in saliva, strengthening its role as a non-invasive medium for integrating dietary exposures, microbial dysbiosis, and host inflammatory or metabolic responses [112]. This multidimensional perspective underscores the value of saliva not only as a reflection of local oral ecology but also as a window into systemic nutritional and metabolic adaptations.

Table 1 synthesizes evidence from population-based studies investigating how dietary exposures, ranging from macronutrient intake, sugar consumption, and meal timing to adherence to specific dietary patterns, modulate salivary biomarkers, oral microbial composition, and clinical oral health indicators across diverse demographic and geographic contexts. The included studies span children, adolescents, adults, and older populations, covering both healthy individuals and groups with specific conditions (e.g., obesity, type 2 diabetes, celiac disease). Reported salivary biomarkers include pH, flow rate, microbial diversity, inflammatory markers, metabolic compounds, and fatty acid profiles. Collectively, these findings highlight the capacity of dietary habits to alter oral and salivary biology, supporting the use of saliva as a non-invasive tool for monitoring diet–microbiome–health interactions.

## 7. Conclusions

Diet and environmental exposures exert profound and multidirectional effects on the oral microbiota, shaping not only local ecological dynamics but also broader metabolic and inflammatory responses with systemic relevance. The evidence reviewed demonstrates that dietary patterns rich in sugars and fermentable carbohydrates promote microbial dysbiosis, acidogenic metabolic activity, and reduced microbial diversity, whereas fiber-rich, plant-based and antioxidant-rich diets support a more balanced, health-associated oral ecosystem. At the same time, exposure to toxic heavy metals, including cadmium, lead, mercury, nickel and arsenic, emerges as an underappreciated disruptor of oral microbial homeostasis, capable of altering microbial structure, promoting inflammatory signaling, and impairing metabolic regulation even at low concentrations.

Saliva has proven to be a highly informative, non-invasive biological matrix that captures these complex interactions. Its composition integrates dietary inputs, microbial metabolic activity, host inflammatory responses and environmental toxicant exposure, making it a promising tool for early detection of nutritional imbalances and metabolic disturbances. Salivary biomarkers ranging from metabolites and oxidative stress indicators to inflammatory cytokines, trace metals and microbial signatures offer unique potential for real-time monitoring of diet–microbiome interactions and individualized metabolic risk. However, despite its promise, the translational implementation of saliva-based diagnostics remains limited by methodological heterogeneity, lack of standardized collection and analytical protocols, and insufficient longitudinal validation.

Overall, the integration of diet, oral microbiota composition, and salivary biomarkers offers a powerful conceptual and methodological framework for understanding how nutritional and environmental factors shape metabolic health. Strengthening this triadic model will advance early prevention, personalized nutritional interventions, and a deeper mechanistic understanding of the oral cavity as a sentinel for systemic well-being.

## Figures and Tables

**Table 1:** Studies Evaluating the Impact of Dietary Factors on Salivary Biomarkers and Oral Health Outcomes.

Country/Population	Dietary Variables Assessed	Oral/Salivary Biomarkers	Main Findings	References
**Turkish adolescents (n = 40)**	Healthy Eating Index (HEI)	DMFT, CPITN, salivary microbiota diversity	Higher diet quality → ↑ microbial diversity; obesity → ↓ diversity; certain phyla correlated with fruit and whole grain intake	[113]
**Australia; healthy caries-free adults (n = 93, age 18–85**	Energy intake, water intake, carbohydrate intake, sugar intake	Salivary flow rate, salivary pH (post-glucose), past caries experience (MF score)	High carb & sugar intake → reduced α-diversity (*p* < 0.05); high sugar intake correlated with higher *Streptococcus* abundance and lower salivary pH; diet explained ~11–12% of microbial variation	[114]
**Spain; adults (n = 40) and children (n = 40) divided into active dentin caries (ADC) and non-ADC groups**	Not specifically diet-focused (mainly caries status)	Salivary flow rate (0.6 ± 0.3 mL/min adults, 0.6 ± 0.4 mL/min children), salivary pH (~7.0 adults, ~7.1 children), total protein content & total antioxidant capacity	No significant differences in salivary biochemical parameters (flow, pH, TPC, TAC) by ADC status; At genus/species level: in adults ADC positively correlated with genera Corynebacterium, Porphyromonas, Tannerella, Filifactor, Dialister etc., and negatively with Haemophilus; in children fewer associations (only Scardovia positive)	[115]
**Spain; older adults (nested case–control, n ≈ 121, age 67–84) affected by T2DM**	Adherence to Mediterranean diet (MEDAS score); consumption of sugary snacks, fish/shellfish, nuts	Salivary microbiome richness & diversity; salivary flow rate; “salivatypes” (microbial community signatures)	Individuals with type-2 diabetes mellitus (T2DM) had lower richness/diversity; three “salivatypes” identified: Salivatype1 (↑ *Streptococcus*, *Veillonella*, *Rothia*) prevalent in T2DM; Salivatype2 (↑ *Prevotella*, *Actinomyces*) associated with obesity; Salivatype3 more common in non-T2DM, non-obese. Salivatype1 associated with higher sugary snack consumption + lower fish/nuts.	[116]
**Finland; children aged 11–13 (n = 453)**	Consumption of “sweet treats” (chocolates/sweets, ice cream, sweet pastries, sugary juice & soft drinks)	Saliva microbiota composition & functional pathways (no classic clinical oral biomarkers)	No difference in alpha-diversity between low vs. high sweet treat groups; significant difference in beta-diversity (*p* = 0.001); higher abundance of *Streptococcus, Prevotella*, *Veillonella*, *Selenomonas* in high sweet treat group (*p* < 0.05).	[117]
**USA; adults, population-based cohorts (n = 989 across two cohorts)**	High-sugar beverage (HSB) intake (low < 1 serving/week; medium 1–3; high > 3)	Saliva/mouthwash microbial community richness & composition	High HSB consumers showed lower species richness (*p* = 0.027) and distinct community profiles (PERMANOVA *p* = 0.040). Commensal bacteria (*Lachnospiraceae*, *Peptostreptococcaceae*, *Alloprevotella rava*) were less common; acidogenic bacteria (*Bifidobacteriaceae*, *Lactobacillus rhamnosus*) more common. No significant interaction with diabetic status or classical caries/periodontitis markers.	[118]
**Italy; young athletes (n = 120) vs. controls (n = 30)**	Number of daily meals; use/frequency of supplements/energy drinks, fruit/juice, snacks/chocolate; difference between usual diet and pre-competition diet	DMFT index; Plaque Index (PI); Gingival Index (GI); salivary resting pH at baseline, before and after training; counts of *Streptococcus mutans* and *Lactobacillus* spp.	Athletes (especially water polo players) showed higher prevalence of caries, erosions, dental stains; energy snacks/chocolate intake strongly associated with the ratio *S. mutans*/*Lactobacillus* spp. (R = 0.88) and with dental defects. Salivary pH differed significantly across observations.	[119]
**Saharawi celiac children (living in refugee camps, then moved to Italy), n = 14 (median age ~8.4 ± 0.7 yrs)**	Dietary intervention: African-style GFD → Italian-style GFD for 60 days Change in dietary pattern: from African-style gluten-free diet (high in cereals, legumes, fiber, low animal protein) to Italian-style gluten-free diet (higher animal proteins, fats, sugars, starch)	Salivary microbiota diversity; relative abundance of bacterial phyla & genera; salivary volatile organic compounds (metabolome)	After switch to Italian-style diet: microbial diversity decreased (*p* < 0.05); shifts: ↓ Firmicutes/Actinobacteria/Tenericutes, ↑ Proteobacteria (*p* = 0.00041); increase in genera Porphyromonas, Neisseria, Granulicatella; decrease in Prevotella, Veillonella; VOC profiles changed (ketones, acids) indicating altered salivary metabolome.	[120]
**Argentina; children aged 6–14 years (n = 45; 20 undernourished, 25 eutrophic)**	Nutritional status (undernourished vs. eutrophic)	DMF/dmf indexes; Plaque Index; Bleeding on probing; Saliva sample	No significant difference in clinical gingival indexes by nutrition status (*p* > 0.05); Undernourished children showed significantly higher prevalence of certain oral microorganisms (*S. gordonii* *p* < 0.05; *Capnocytophaga gingivalis* *p* < 0.01; *Fusobacterium nucleatum p* < 0.05; etc.)	[121]
**Germany; non-obese men (n = 29; age 20–40)**	Meal timing: high-carb breakfast + high-fat dinner vs. high-fat breakfast + high-carb dinner (isocaloric diets)	Salivary cortisol, melatonin, resistin, adiponectin, IL-6, MCP-1 (24 h profiles)	Salivary biomarkers showed clear diurnal rhythms and strong correlations with blood levels. Meal timing (carb–fat distribution) had minimal effect on metabolic or inflammatory salivary biomarker patterns.	[122]

Arrows indicate the direction of change: ↑ increase, ↓ decrease, → association.

## Data Availability

No new data were created or analyzed in this narrative review. Data sharing is not applicable to this article.
